# The Phosphodiesterase Inhibitor Tadalafil Promotes Splenic Retention *of Plasmodium falciparum* Gametocytes in Humanized Mice

**DOI:** 10.3389/fcimb.2022.883759

**Published:** 2022-05-25

**Authors:** Daniela Barbieri, Lina Gomez, Ludivine Royer, Florian Dupuy, Jean-François Franetich, Maurel Tefit, Marie-Esther N’Dri, Dominique Mazier, Olivier Silvie, Alicia Moreno-Sabater, Catherine Lavazec

**Affiliations:** ^1^INSERM U1016, CNRS UMR8104, Université Paris Cité, Institut Cochin, Paris, France; ^2^Sorbonne Université, INSERM, CNRS, Centre d’Immunologie et des Maladies Infectieuses, Paris, France; ^3^Service de Parasitologie-Mycologie Assistance Publique-Hôpitaux de Paris (AP-HP), Hôpital Saint-Antoine, Paris, France

**Keywords:** *Plasmodium falciparum*, transmission, gametocytes, tadalafil, phosphodiesterase, humanized mice

## Abstract

The persistence of erythrocytes infected with *Plasmodium falciparum* gametocytes in the bloodstream is closely related to the modulation of their mechanical properties. New drugs that increase the stiffness of infected erythrocytes may thus represent a novel approach to block malaria parasite transmission. The phosphodiesterase inhibitor tadalafil has been shown to impair the ability of infected erythrocytes to circulate in an *in vitro* model for splenic retention. Here, we used a humanized mouse model to address *in vivo* the effect of tadalafil on the circulation kinetics of mature gametocyte-infected erythrocytes. We show that stiff immature gametocyte-infected erythrocytes are retained in the spleen of humanized mice at rates comparable to that of the *in vitro* model. Accordingly, tadalafil-induced stiffening of mature gametocyte-infected erythrocytes impairs their circulation in the bloodstream and triggers their retention by the spleen. These *in vivo* results validate that tadalafil is a novel drug lead potentially capable of blocking malaria parasite transmission by targeting GIE mechanical properties.

## Introduction

Malaria is a major public health problem that still causes more than half a million deaths per year (WHO, 2021). Clinical symptoms of malaria are ascribed to the asexual stages of the *Plasmodium* parasite, while gametocytes, the specialized sexual cells, are responsible for parasite transmission from humans to Anopheles mosquitoes. Most antimalarials target the asexual stages, and are less efficient in killing sexual stages (Birkholtz et al., 2016; Plouffe et al., 2016). This poses a significant challenge to malaria elimination strategies because infected individuals could still transmit the sexual parasites even several weeks after successful treatment of the asexual forms (Bousema et al., 2006). Thus, the development of novel compounds is urgently needed in order to target gametocytes. *Plasmodium falciparum* gametocytes require 8-10 days for maturation into five morphologically distinct phases (stages I-V) (Hawking et al., 1971). Stages I-IV are absent from blood circulation and are instead sequestered in the bone marrow where they develop into mature stage V gametocytes (Farfour et al., 2012; Aguilar et al., 2014; Joice et al., 2014). Mature gametocyte-infected erythrocytes (GIE) are then released in the blood, infective for mosquitoes. The lifespan of circulating mature gametocytes has been estimated at several weeks during which stage V GIE are able to avoid splenic retention (Bousema et al., 2010; Cao et al., 2019). GIE persistence in the blood circulation is crucial for parasite transmission to mosquitoes; therefore, interfering with this process is an innovative approach to block malaria parasites transmission (Tiburcio et al., 2012; Duez et al., 2015). The filterability of mature GIE is closely related to the modulation of their mechanical properties. During their development, gametocytes decrease both the stiffness and the permeability of their host erythrocyte, thus increasing its filterability across the spleen and its osmotic stability (Aingaran et al., 2012; Dearnley et al., 2012; Tiburcio et al., 2012; Bouyer et al., 2020). These modifications of GIE mechanical properties are tightly regulated by cyclic AMP (cAMP) signaling pathway and phosphodiesterase (PDE) activity (Ramdani et al., 2015; Bouyer et al., 2020). *Pf*PDEδ, the main PDE expressed by mature gametocyte stages, plays a key role in this process and appears to be the master regulator of mature GIE deformability (Ramdani et al., 2015). Importantly, studies using an *in vitro* model for splenic retention reported that the marketed PDE inhibitors sildenafil and tadalafil increase the stiffness of mature GIE (Ramdani et al., 2015; N’dri et al., 2021). These observations suggest that drugs targeting PDE are expected to promote the mechanical retention of mature GIE by the spleen and their clearance from the bloodstream in gametocyte carriers. PDE inhibitors therefore represent potential novel drug leads capable of blocking malaria parasite transmission. To reinforce this drug development effort, these promising observations should be validated *in vivo*. The effect of PDE inhibitors on the circulation of *Plasmodium* gametocytes was investigated using the rodent model *Plasmodium berghei* in a study showing that treatment of infected mice with sildenafil citrate results in gametocyte accumulation in bone marrow and spleen (De Niz et al., 2018). However, since *P. falciparum* parasites are highly specific for human red blood cells (hRBC) and exhibit a sexual development drastically different from that of rodent malaria parasites, the use of a humanized mouse model is the most relevant approach to validate the effect of PDE inhibitors *in vivo* (Moreno-Sabater et al., 2018). To this end, we used mice from the severe immunodeficient mouse strain NOD SCID gamma c (NSG) in which we induced a chemical depletion of macrophages and neutrophils. In these mice, the immunomodulation protocol allows the graft of hRBC and prevents the elimination of GIE by the immune system, thus making this humanized mouse model suitable for testing drugs against *P. falciparum* transmission stages (Duffier et al., 2016).

In this study, we used flow cytometry, bioluminescence imaging and RT-qPCR analyses to follow the kinetics of *P. falciparum* GIE in the blood circulation of this humanized mouse model over a short or a long period of time in order to quantify the effect of tadalafil on GIE persistence in the bloodstream and retention in the spleen.

## Material and Methods

### Parasites and Mice

The *P. falciparum* NF54 strain, the transgenic lines NF54-cg6-pfs16-CBG99 (Cevenini et al., 2014), NF54-pfs47-hsp70-GFP (Neveu et al., 2020) and NF54-hsp70-TurboFP635 (described in **Supplemental Methods**) have been used for our experiments. Parasites were cultured *in vitro* under standard conditions using RPMI-1640 medium supplemented with 10% heat-inactivated human serum and human erythrocytes at a 5% hematocrit. Synchronous production of specific gametocytes stages was achieved by treating synchronized cultures at the ring stage (10-15% parasitemia) with 50 mM N-acetylglucosamine (NAG) for 5 days to eliminate asexual parasites. Gametocytes were purified by magnetic isolation using a MACS depletion column (Miltenyi Biotec) in conjunction with a magnetic separator.

NSG mice were purchased from Charles River. All animal experiments were carried out in strict accordance with the guide for the care and use of laboratory animals from the Centre d’Expérimentation Fonctionnelle (CEF, La Pitié-Salpêtrière, Paris) and with the French and European regulations (2010/63/EU). The experimental protocols were approved by the Ministère de l′Education Nationale, de l’Enseignement Supérieur et de la Recherche (Authorization Number 01736.02). For all tests, 9–12 weeks-old mice were used. Infection experiments were performed using 10-15 animals per experiment, while GIE injections were performed using 1-6 animals per experiment.

### Immunomodulation Treatment and hRBC Engraftment in Mice

NSG mice aged 9-12 weeks were bred at the CEF under strict pathogen-free conditions. hRBC were obtained from donors (Etablissement Français du Sang Ile-de-France, Rungis). Before peritoneal injection into mice, hRBC were washed twice with RPMI-1640 medium at 2500 rpm, 5 min at 4°C. Immunomodulation treatment was performed as described in (Duffier et al., 2016). The depletion of tissue macrophages was induced by clodronate encapsulated in liposomes (Liposoma). Neutrophils were depleted using the monoclonal antibody (mAb) NIMP-R14 produced by a hybridoma kindly provided by Dr M. Strath (National Institute for Medical Research, London, U.K.) (Tacchini-Cottier et al., 2000). To obtain the graft of hRBC and subsequent *P. falciparum* infection, each mouse received by intraperitoneal injection a dose of 1 mg/kg of mAb NIMP-R14 at day 0 and 6.25 mg/kg of lip-clod at day 1. At day 5 and day 7 each mouse received 1.5 mL of hRBC at 50% hematocrit in RPMI mixed with 1 mg/kg of mAb NIMP-R14 and 6.25 mg/kg of lip-clod. At day 9, mice were subjected to either infection with asexual parasites or injection of purified GIE. Hematological parameters (hematocrit, leucocytes, platelets, percentage of hRBC in peripheral blood) were followed up during the assay in blood samples taken from mouse tails and analyzed with an automatic hematology analyzer. Mice with incomplete hRBC engraftment at day 9 were excluded from subsequent analyses.

### Mouse Infection Using Asexual Parasites

At day 9 after the beginning of immunomodulation treatment, each mouse received the same doses of immunomodulators in 1.5 mL of hRBC at 50% hematocrit containing 0.1% of NF54-pfs47-hsp70-GFP *P. falciparum* asexual parasites. Parasites used for infection were obtained from an *in vitro* culture maintained below 1.5% parasitemia to avoid *in vitro* induction of gametocytogenesis. After *P. falciparum* infection, mice were grafted with 1 to 1.5 mL of hRBC at 50% hematocrit containing 1 mg/kg of mAb NIMP-R14 and 6.25 mg/kg of lip-clod every 2–3 days. Mice with hematocrit above 60% and percentage of hRBC above 70% received the graft of hRBC only when hematocrit decreased to 50%.

### Mouse Injection Using GIE

At day 9 after the beginning of immunomodulation treatment, each mouse received 6x10^7^ *P. falciparum* GIE by retro-orbital injection. GIE preparations were enriched in different experiments by magnetic isolation. Stage III GIE were collected at day 4 and stage V GIE at day 9 after initiating NAG treatment. Parasites were kept at 37°C until injection to avoid activation of gametogenesis.

### Tadalafil Treatment

The effect of tadalafil was assessed either upon incubation of purified GIE or upon mice treatment. In the first instance, MACS-purified stage V GIE preparations were pre-incubated 30 minutes at 37°C in RPMI-1640 medium supplemented with 100 µM tadalafil (Euromedex) before retro-orbital injection of 100 µl of RPMI/talalafil 100µM in each mouse. Treatment of mice with tadalafil was administered either to uninfected mice before GIE injection or to infected mice when gametocytes were circulating in peripheral blood for more than 1 day. Uninfected mice were orally treated with a dose of 200 µg Cialis^®^ (Lilly) diluted in 0.9% NaCl, which corresponds to a tadalafil dose of 8 mg/kg, 30 minutes before retro-orbital injection with MACS-purified stage V GIE. This dose was chosen based on the interspecies dose extrapolation scaling to result in plasma concentrations of tadalafil equivalent to a human dose of 40 mg/day. In mouse infection experiments, 200 µg of Cialis^®^ (Lilly) diluted in 0.9% NaCl was administered by oral feeding daily during 4 days. For each mouse, gametocytemia was monitored on Giemsa-stained thin tail-blood smears collected daily until animal sacrifice at day 6 post drug treatment and by RT-qPCR analysis on blood collected at sacrifice.

### Quantification of Parasitemia by Flow Cytometry

After GIE injection, the quantification of parasitemia in mouse peripheral blood was performed using flow cytometry. To increase the fluorescent signal, GIE NF54-pfs47-hsp70-GFP and NF54-hsp70-TurboFP635 were pre-incubated for 15 minutes at 37°C with WGA-GFP or WGA-PE Texas red (5 µg/mL), respectively. Five minutes before the end of incubation, GIE were stained with Hoechst 33342 (1/10,000). Cells were then washed and resuspended in 100 μL RPMI-1640 before retro-orbital injection in mice. Parasitemia was followed up during the assay in blood samples taken from mouse tails at 10 minutes, 1 hour, 2 hours, 3 hours and 7 hours post GIE injection. The collected samples (100 µL of peripheral blood) at each time point were washed with PBS 1 X and fixed for 10 min at room temperature with PBS, 1% PFA and 0.025% Glutaraldehyde. Parasitemia was quantified using Fortessa (BD Biosciences) cytometer.

### Quantification of Parasitemia by RT-qPCR Analysis

Peripheral blood samples were added to Trizol (Life technologies) and vortexed, while spleen samples were grinded in Trizol. RNA was prepared using the PureLink RNA Mini kit (Life technologies) and treated using on-column DNase-Treatment with Pure Link DNase (Life technologies). Quantity and purity of RNA were assessed with Nanodrop 8000 (Thermo Scientific). cDNA synthesis was performed using the SuperScript III First-Strand Synthesis System (Life technologies). Different sets of primers were used to quantify parasites: when two transgenic parasite lines were co-injected in mice, primers designed for *gfp* (*sense 5’-*TTCTTCAAGTCCGCCATGCC*, antisense 5’-*TTGTACTCCAGCTTGTGCCC) or *turbofp635* (*sense 5’-*CAAAACCTTTATCAACCACACC, *antisense 5’-* CCGAGTGTTTTCTTCTGCATC) were used to discriminate between the two subpopulations of co-injected gametocytes (stage III vs stage V or tadalafil-treated vs untreated), while primers for the ubiquitin-conjugating enzyme (*HK*, *PF3D7_0812600, sense 5’-*GGTGTTAGTGGCTCACCAATAGGA*, antisense 5’-*GTACCACCTTCCCATGGAGTATCA) were used to quantify the whole population of gametocytes. When only one transgenic parasite line was injected in mice, parasitemia was quantified using the absolute quantification method by determining a standard curve after amplification of the *gfp* sequence from serial dilutions of the pBLD588-hsp70-GFP plasmid (Neveu et al., 2020). qPCRs were performed in Light Cycler 480 (Roche), each sample was analyzed in duplicates.

### Assessment of GIE Localization *In Vivo*

GIE distribution in mouse tissue was determined by bioluminescence imaging. Mice were retro-orbitally injected with 6x10^7^ NF54-cg6-pfs16-CBG99 GIE. Seven hours post-injection, mice were injected intraperitoneally with D-luciferin (potassium salt, Perkin Elmer) at 100 mg/kg, sacrificed 3 minutes after injection, dissected and then organs (brain, heart, lung, liver, spleen and bone) were imaged within 10-15 min post-injection. IVIS Spectrum (Caliper Life Science, Hanover, MD, USA) was used to measure luciferase activity. Images were analyzed using the living Image 3.0 software (Capiler Life Science, Hanover, MD, USA). The luminescence signal was measured in photons s^-1^ cm^-2^ sr^-1^.

### Microsphiltration

Calibrated metal microspheres (96.50% tin, 3.00% silver, and 0.50% copper; Industrie des Poudres Sphériques) with 2 different size distributions (5- to 15-μm-diameter and 15- to 25-μm-diameter) composed a matrix used to assay infected erythrocyte deformability under flow, as described (Lavazec et al., 2013). Suspensions of cultures containing 1.5% of stage III or stage V GIEs were perfused through the microsphere matrix at a flow rate of 60 mL/h using an electric pump (Syramed_sp6000, Arcomed_Ag), followed by a wash with 5 mL of complete medium. The upstream and downstream samples were collected and smeared onto glass slides for staining with Giemsa reagent, and parasitemia was assayed by counting 2000 erythrocytes to determine parasite retention versus flow-through.

### Generation of the *NF54*-Pfs47-hsp70-TurboFP635 Transgenic Line

To generate the NF54-pfs47-hsp70-TurboFP635 line that expresses the far-red reporter TurboFP635 under the control of the constitutive promoter *hsp70*, cultures of the NF54 clone B10 were co-transfected with 70 μg of plasmid pDC2-Cas9-hDHFRyFCU and 70 μg of plasmid pBLD588-hsp70-TurboFP635 and selected with 2.5 nM WR99210 as previously described (Ghorbal et al., 2014). The plasmid pDC2-Cas9-hDHFRyFCU (Knuepfer et al., 2017) encodes a single guide RNA that recognizes a sequence located in the *pfs47* locus. Generation of pBLD588-hsp70-TurboFP635 plasmid was performed by a double digestion of the pBLD588-hsp70-GFP plasmid previously described (Neveu et al., 2020) with HindIII and XhoI restriction enzymes. The *turbofp635* was amplified from a gBlocks Gene Fragments (IDT) using the P2 (TTAAGAAAAAAAGCTTATGGTGGGTGAGGATAGCGTGC) and P4 (CGTTATGTTACTCGAGTTAGCTGTGCCCCAGTTTGCTAGG) primers. The *turbofp635* fragment was then cloned in frame using the In-Fusion system (Ozyme). Following transfection and drug selection, clones were obtained by limiting dilution. Plasmid integration into the *pfs47* locus was confirmed by PCR using the primers P3 (GCGATATGTAATTCCATTACTGC) and P1 (CCTAACACATTATGTGTATAACATTTTATGC).

### Statistical Analysis

Statistical significance was determined using a Mann Whitney test or a two-way ANOVA test with Sidak correction for multiple comparisons. Analyses were performed using GraphPad Prism Version 9.3.1 for Windows.

## Results

### Stiff GIE Are Cleared Faster From the Peripheral Circulation Than Deformable GIE

**Figure 1:**
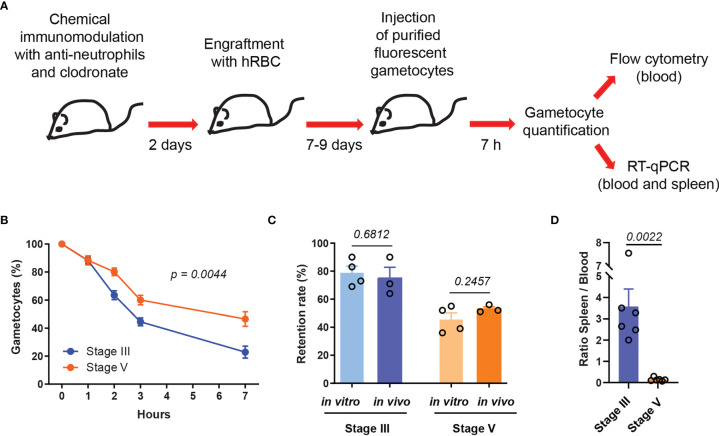
Stiff immature GIE are cleared faster from the peripheral circulation than deformable mature GIE. **(A)** Schematic of experimental procedure for immunomodulation treatment and hRBC engraftment of NSG mice before injection with *P. falciparum* immature (stage III) and mature (stage V) fluorescent gametocytes. **(B)** Quantification of GIE clearance in peripheral blood, comparing stage III GIE (blue) and stage V GIE (orange) by flow cytometry (n = 6 mice from 2 independent experiments). The percentage of gametocytes is normalized to the gametocytemia at 10 minutes after injection. *p* indicates the statistical significance determined using a two-way ANOVA test. **(C)** Retention rate of stage III and stage V GIE determined *in vitro* by microsphiltration (n = 4 filtration columns) and *in vivo* at 7hpi by flow cytometry (n = 3 mice). To determine GIE retention *in vitro* the upstream and downstream samples were smeared, stained with Giemsa and parasitemia was assayed by counting at least 2000 erythrocytes. **(D)** Quantitative analysis by real time RT-qPCR of GIE distribution in peripheral blood and spleen. Results were calculated as relative copy number of *gfp* or *turbofp635* gene transcripts to the control housekeeping *HK* gene transcripts in spleen and blood samples. The graph represents the ratio spleen-to-blood for 6 mice from 2 independent experiments. In C and D, *p* indicates the statistical significance determined using a Mann Whitney test. Error bars show the standard error of the mean (SEM).

To analyze the kinetics of *P. falciparum* GIE circulation *in vivo*, we used chemically immunomodulated NSG mice grafted with hRBC (Duffier et al., 2016). After successful hRBC engraftment, MACS-purified fluorescent GIE were retro-orbitally injected into mice and percentage of GIE was then monitored by flow cytometry in blood collected from the tail of the mice at several time points from 10 minutes post-injection until sacrifice. Preliminary results showed that almost all mature GIE injected in mice were cleared from the peripheral blood at 24 hours post injection (hpi), whereas approximately half of the GIE population remained in circulation at 7 hpi (**Supplemental Figure 1**). Therefore, in the following experiments all mice were sacrificed at 7 hpi. To address the impact of GIE deformability on their circulation kinetics, stiff immature GIE and deformable mature GIE from two different fluorescent transgenic strains, which constitutively express either a green fluorescent reporter [NF54-Pfs47-hsp70-GFP (Neveu et al., 2020)] or a far red fluorescent reporter [NF54-Pfs47-hsp70-TurboFP635 (**Supplemental Figure 2**)], were simultaneously injected into each mouse (**Figure 1A**). This protocol allows to follow two populations of parasites in the same mouse by flow cytometry or by RT-qPCR using primers designed on the fluorescent reporter sequences. To avoid parasite strain effect, three mice were co-injected with 6x10^7^ immature GFP-expressing GIE and 6x10^7^ mature TurboFP635-expressing GIE, whereas three other mice were co-injected with 6x10^7^ immature TurboFP635-expressing GIE and 6x10^7^ mature GFP-expressing GIE. Flow cytometry analysis showed that about 50% of the mature GIE population disappeared from the peripheral blood in 6 hours whereas 3 hours were sufficient to observe the clearance of half of the immature GIE population. At 7 hpi, only 22% of immature GIE persisted in the peripheral blood compared to 47% of mature GIE, indicating that stiff GIE are cleared faster from the circulation than deformable GIE (**Figure 1B**). Interestingly, these proportions were comparable to the retention rates observed with the same parasites in an *in vitro* model for splenic retention (**Figure 1C**). These observations led to the hypothesis that clearance of GIE from the peripheral blood may result from retention in the mouse spleen. To address this hypothesis, we performed RT-qPCR on blood and spleen samples collected from mice sacrificed at 7 hpi, using primers designed on the fluorescent reporter sequences to discriminate between immature and mature GIE within the same sample. The RT-qPCR analysis showed that the ratio spleen-to-blood was 25-fold higher for stage III GIE than for stage V GIE (**Figure 1D**). These results indicate that immature GIE, which are stiff, persist less long in the peripheral blood and are retained more in the spleen of humanized mice than mature GIE, which are deformable.

### Stiff GIE Are Retained in the Spleen of Humanized Mice

**Figure 2:**
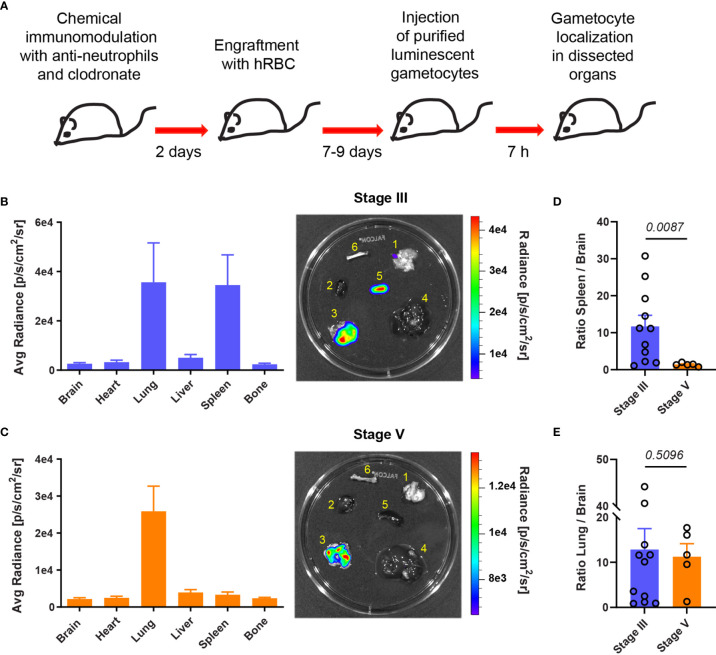
Stiff immature GIE are retained in the spleen. **(A)** Schematic of experimental procedure for immunomodulation treatment and hRBC engraftment of NSG mice before injection with luciferase-expressing immature (stage III) and mature (stage V) gametocytes. GIE distribution was quantified 7 hpi by measuring luciferase activity in dissected organs. **(B, C)** Left: Quantification of GIE distribution visualized by measuring average radiance (p/s/cm^2^/s) in dissected organs (B: stage III n=11 mice from 4 independent experiments; C: stage V n=5 mice from 2 independent experiments). Right: Representative images of luminescent signals in dissected organs (1, brain; 2, heart; 3, lungs; 4, liver; 5, spleen; and 6, bone) and heat map of radiance (p/s/cm^2^/sr). Rainbow shows the relative level of luciferase activity. Note that the scale of radiance can be different within separate illustration. **(D, E)** Quantification of luciferase activity calculated as the ratio of radiance in spleen and brain **(D)** or in lung and brain **(E)**. Circles indicate the number of mice from four independent experiments for stage III GIE and two independents experiments for stage V GIE. *p* indicates the statistical significance determined using a Mann Whitney test. Error bars show the SEM.

To analyze further the retention of GIE in different mouse organs, mice were injected with luciferase-expressing GIE from the NF54-cg6-pfs16-CBG99 strain (Cevenini et al., 2014). Immature and mature GIE localization was assessed at 7 hpi by quantifying bioluminescence in dissected organs (**Figure 2A**). Upon injection of immature GIE, measurement of luminescence signals in isolated organs revealed GIE accumulation in the lungs and the spleen (**Figure 2B**). In contrast, injection of mature GIE resulted in a much lower luminescence signal in the spleen (**Figures 2C, D**). The signal in the lungs was comparable after injection of both stages (**Figure 2E**), suggesting that accumulation of parasites in the lungs in not dependent of GIE deformability. These results confirm that stiff GIE are retained more in the spleen of humanized mice than deformable GIE. Taken together, these findings indicate that this mouse model can be used to test drugs that interfere with the mechanical properties of GIE.

### Pre-Incubation of Mature GIE With Tadalafil Induces Their Retention in the Spleen

**Figure 3:**
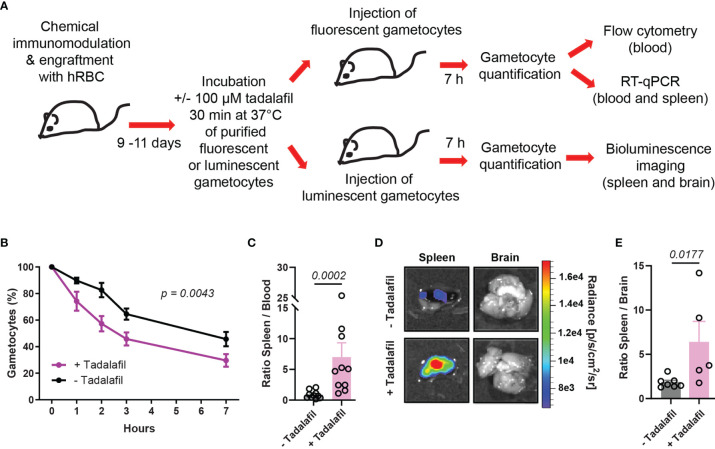
Pre-incubation of mature GIE with tadalafil induces their retention in the spleen. **(A)** Schematic of experimental procedure for immunomodulation treatment and hRBC engraftment of NSG mice before injection with tadalafil-treated or untreated GIE. Stage V GIE were pre-incubated or not 30 min at 37°C with 100 µM tadalafil before injection in mice. At 7 hpi, quantification of fluorescent GIE was performed by flow cytometry and RT-qPCR while quantification of luciferase-expressing GIE in spleen was performed by measuring the bioluminescence signal. **(B)** Quantification of GIE clearance in peripheral blood by flow cytometry (n = 10 mice from 5 independent experiments). The percentage of gametocytes is normalized to the gametocytemia at 10 minutes after injection. *p* indicates the statistical significance determined using a two-way ANOVA test. **(C)** Quantitative analysis by real time RT-qPCR of GIE distribution in peripheral blood and spleen. Results were calculated as relative copy number of *gfp* or *turbofp635* gene transcripts to the *HK* gene transcripts in spleen and blood samples. The graph represents the ratio spleen-to-blood for 10 mice from 5 independent experiments. **(D)** Representative image of luminescence signal in dissected spleen and brain from mice injected with tadalafil-treated and untreated GIE. The brain was used as negative control. **(E)** Quantification of luciferase activity calculated as the ratio of radiance in spleen and brain from mice injected with tadalafil-treated and untreated GIE. Circles indicate the number of mice from 5 independent experiments for untreated GIE and from 4 independent experiments for tadalafil-treated GIE. In C and E, *p* indicates the statistical significance determined using a Mann Whitney test. Error bars show the standard error of the mean (SEM).

To address the effect of tadalafil treatment on GIE circulation *in vivo*, mature GIE were first pretreated *in vitro* before their injection in humanized mice. 6x10^7^ GFP-expressing and 6x10^7^ TurboFP635-expressing mature GIE were MACS-purified and one of these two transgenic lines was pre-incubated 30 min *in vitro* with 100 µM tadalafil. The persistence of treated and untreated GIE in the peripheral blood was then analyzed by flow cytometry during 7 hours (**Figure 3A**). As observed for immature stages, tadalafil-treated mature GIE were cleared faster from the peripheral blood than control GIE, with half of treated GIE disappearing in less than 3 hours versus 6 hours for untreated GIE. At 7hpi, 71% of the initial tadalafil-treated GIE population was cleared from the peripheral blood of mice (**Figure 3B**). RT-qPCR analysis was performed to further quantify the distribution of control and treated GIE in the spleen and in the peripheral blood at 7 hpi. The ratio spleen-to-blood was 8-fold higher for treated GIE than for control GIE, confirming that tadalafil-treated GIE persist less in blood circulation than untreated GIE and are retained more in the spleen (**Figure 3C**). These conclusions were strengthened by quantification of luminescence signals in the spleen after injection of luminescent GIE. Seven hours after injection of 6x10^7^ untreated mature GIE expressing luciferase, the luminescence signal in the spleen was very weak, however the signal was much higher when mice were injected with mature GIE pre-incubated with 100 µM tadalafil (**Figures 3D, E**). These results show that chemically-induced stiffening of mature GIE induces their retention in the spleen *in vivo* and therefore validate our previous results observed in an *in vitro* model for splenic retention (Ramdani et al., 2015; N’dri et al., 2021).

### Circulation of Mature GIE Is Impaired in Humanized Mice Orally Treated With Tadalafil

**Figure 4:**
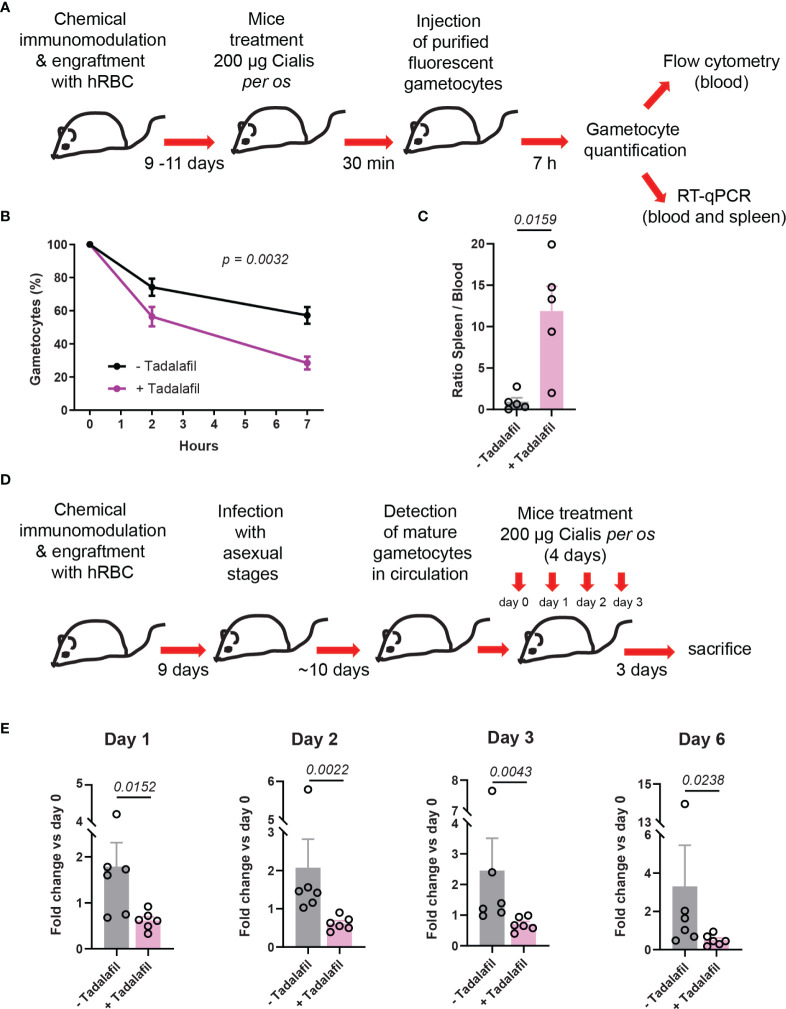
Circulation of mature GIE is decreased in humanized mice treated orally with tadalafil. **(A)** Schematic of experimental procedure for immunomodulation treatment and hRBC engraftment of NSG mice before injection with NF54-pfs47-hsp70-GFP stage V GIE. Mice were orally treated with Cialis 30 min before stage V GIE injection. GIE quantification was performed at 7 hpi by flow cytometry and RT-qPCR. **(B)** Quantification of stage V GIE clearance in peripheral blood, comparing tadalafil-treated (violet) and untreated mice (black) by flow cytometry (n = 5 mice from 3 independent experiments). The percentage of gametocytes is normalized to the gametocytemia at 10 minutes after injection. *p* indicates the statistical significance determined using a two-way ANOVA test. **(C)** Quantitative analysis by real time RT-qPCR of GIE distribution in peripheral blood and spleen. Results were calculated as *gfp* transcript copy number using the absolute quantification method. Circles indicate the number of mice from 3 independent experiments. *p* indicates the statistical significance determined using a Mann Whitney test. **(D)** Experimental procedure for immunomodulation treatment and hRBC engraftment of NSG mice before infection with *P. faciparum* asexual parasites. Mice were treated orally with Cialis during 4 days and gametocytemia was monitored in thin tail-blood smears collected daily until sacrifice at day 6 after initiating drug treatment (n = 6 mice from 2 independent experiments). **(E)** Quantification stage V GIE in circulation at day 1, day 2, day 3 and day 6 after initiating the treatment. Gametocytemia was determined by counting 2000 to 4000 erythrocytes on thin tail-blood smears. *p* indicates the statistical significance determined using a Mann Whitney test. Circles indicate the number of mice from 2 independent experiments. Error bars show the SEM.

To further address the effect of tadalafil on mature GIE circulation *in vivo*, we applied a protocol taking into account the pharmacokinetics of tadalafil. Mice were orally treated with the Cialis^®^ drug, which is the pharmaceutical form of tadalafil. A group of 5 humanized NSG mice were orally treated with 200 µg of Cialis^®^ 30 min before injection of 6x10^7^ purified GFP-expressing mature GIE (**Figure 4A**). At 7 hpi, the clearance of mature GIE in tadalafil-treated mice was significantly increased compared to untreated mice. Flow cytometry analysis revealed that an average of 57% gametocytes persisted in the peripheral blood of untreated mice compared to only 26% in the group of tadalafil-treated mice (**Figure 4B**). Quantification of GIE was then performed by RT-qPCR with primers designed on the *gfp* sequence on blood and spleen samples collected from mice sacrificed at 7 hpi. The RT-qPCR analysis showed that the ratio spleen-to-blood was 13-fold higher in Cialis-treated mice than in untreated mice (**Figure 4C**). Together, these results show that mice treatment with tadalafil leads to a decrease of GIE presence in the circulation and their accumulation in the spleen. To further address the effect of tadalafil over a longer period of time, we used an infection protocol suitable for testing drugs against *P. falciparum* transmission stages (Duffier et al., 2016). In this protocol, NSG mice are humanized before being infected with asexual parasites, and then mature gametocytes are detected in the peripheral circulation after an average of 10 days (**Figure 4D**). After the appearance of stage V gametocytes in peripheral blood, a group of 6 mice were daily treated with 200 µg of Cialis^®^ *per os* for 4 days (from day 0 to day 3). A group of 6 untreated mice was used as a control. For each mouse, gametocytemia was monitored in thin tail-blood Giemsa-stained smears collected daily until sacrifice at day 6 after initiating the drug treatment. Between day 0 and day 3, stage V gametocytemia increased approximately 2-fold in control mice, as a result of the increase of asexual parasitemia that occurred in these mice several days earlier (**Figure 4E**). In contrast, this increase was not observed in Cialis-treated mice in which the gametocytemia slightly decreased. The effect of tadalafil treatment was even more pronounced three days after the end of the treatment (at day 6) when stage V gametocytemia in control mice increased about 3-fold compared to day 0 whereas it decreased 2-fold in treated mice. These results indicate that tadalafil treatment impedes the increase in gametocytemia and promote gametocyte clearance form peripheral circulation.

## Discussion

Targeting the mechanical properties of mature GIE to interfere with their circulation across the spleen has been proposed as a promising strategy to block malaria parasite transmission (Tiburcio et al., 2012; Duez et al., 2015; Russell and Cooke, 2017; Henry et al., 2020). In this context, the PDE inhibitors sildenafil and tadalafil have previously shown their ability to block the filtration of mature GIE in an *in vitro* model of splenic retention (Ramdani et al., 2015; N’dri et al., 2021). In this study, we used a humanized mouse model to address *in vivo* the effect of tadalafil on the circulation of mature GIE. Our results indicate that the retention rates in the humanized mouse spleen are comparable to that observed *in vitro* by microsphiltration, the physiological relevance of which has been validated with *ex vivo* perfused human spleens (Deplaine et al., 2011). Importantly, stiff GIE were retained in the mouse spleen, resulting in faster clearance from the peripheral blood than deformable GIE. Accordingly, upon pretreatment of GIE before injection or upon oral treatment of mice, tadalafil-induced stiffening of mature GIE also impaired their circulation and triggered their retention by the spleen. These results confirm that tadalafil represents a novel drug lead potentially capable of blocking malaria parasite transmission by impacting on GIE circulation. Although this humanized mouse model has previously been used to test the gametocytidal drug primaquine (Duffier et al., 2016), our results indicate that it is also suitable for testing drugs that impact the mechanical properties of infected erythrocytes. Another study reported that calyculin, a phosphatase inhibitor known to stiffen mature GIE (Ramdani et al., 2015), induces rapid elimination of pretreated GIE from the peripheral blood after injection in macrophage-depleted mice (Duez et al., 2015). However, the localization of GIE in mouse organs was not addressed in this study, and in the absence of hRBC graft, it is likely that GIE were sequestered in the liver rather than the spleen, as we observed in ungrafted mice (**Supplemental Figure 3**). In contrast, bioluminescent imaging analyses performed in our study confirmed that the main retention of stiff GIE occurs in the spleen, since every other day injection of hRBC may saturate the liver, which is the major site of hRBC sequestration (Song et al., 2021). Significant accumulation of GIE was also observed in the lungs, although this retention was not dependent on GIE deformability since the bioluminescent signal in the lungs was comparable after injection of either immature or mature GIE. Sequestration of *Plasmodium* parasites in the lungs has previously been observed in mice infected with asexual forms of the murine malaria parasite *P. berghei* (Franke-Fayard et al., 2005; Franke-Fayard et al., 2010; Fonager et al., 2012; De Niz et al., 2016). The class II scavenger-receptor CD36 was identified as the major receptor for *P. berghei* sequestration in this organ, however the parasite ligand(s) is not identified yet (Franke-Fayard et al., 2005). CD36 is a receptor widely distributed on endothelial cells and is a major binding receptor for several *Plasmodium* species, including *P. falciparum* (Ho and White, 1999). Thus, we can speculate that CD36 is involved in the accumulation of GIE observed in the lungs in this study. Our bioluminescent imaging analyses also reveal that injected stiff GIE are not detected in bones, whereas in previous study immature GIE accumulated in the bone marrow of *P. falciparum* infected mice (Duffier et al., 2016). A possible explanation is that immature gametocytes observed in the bone marrow upon infection originated from sexually-committed rings that entered the bone parenchyma and were sequestered there because of their high rigidity, whereas upon injection, the stiffness of infused immature GIE may prevent their entry into the bone marrow parenchyma.

Our results show that splenic retention of GIE upon tadalafil treatment is accompanied by a faster clearance from the peripheral blood, confirming that tadalafil represents a novel drug lead potentially capable of blocking malaria parasite transmission by impacting on GIE circulation. However, even after 4 days of tadalafil treatment, GIE were not completely eliminated from the bloodstream in this mouse model. It should be noted that these mice are depleted in macrophages due to clodronate treatment, therefore the low number of resident splenic macrophages and the reversible effect of tadalafil (Seftel, 2004) may explain why some GIE are not destroyed in the spleen and are released into the bloodstream. It might be of interest to test the effect of tadalafil in a recent humanized mouse model which allows resident tissue macrophages to populate host tissues combined with enhanced reconstitution of human erythropoiesis and mature circulating hRBC (Song et al., 2021). This mouse model would allow to study not only the role of splenic macrophages on stiff GIE destruction but also the effect of drugs on gametocytes that develop in erythroblasts and reticulocytes (Neveu et al., 2020). None the way, we can speculate that in humans, resident splenic macrophages would be able to eliminate mature GIE retained in this organ upon stiffening treatment. As tadalafil is an FDA-approved drug, we may consider testing its effect on the circulation kinetics of *P. falciparum* mature GIE in a controlled model of human malaria infection (Collins et al., 2018; Reuling et al., 2018). In addition, the structure of tadalafil can be easily modified to suppress its effect on human targets and therefore generate *Plasmodium*-specific PDE inhibitors (Beghyn et al., 2011; Beghyn et al., 2012). Further optimization of these compounds through structural modifications could lead to more effective and irreversible inhibitors capable of completely removing gametocytes from the bloodstream.

## Data Availability Statement

The raw data supporting the conclusions of this article will be made available by the authors, without undue reservation.

## Ethics Statement

The animal study was reviewed and approved by Ministère de l′Education Nationale, de l’Enseignement Supérieur et de la Recherche (Authorization Number 01736.02).

## Author contributions

CL and DB conceived the project. DB, LG, LR, and FD performed the experiments. CL and DB designed and interpreted the experiments. J-FF, MT, M-EN, DM, OS, and AM-S contributed resources or data. CL and DB wrote the article. All authors contributed to the article and approved the submitted version.

## Funding

This study was supported by grants from the Fondation pour la Recherche Médicale (“Equipe FRM” grant EQ20170336722 and “Ingenieur FRM” grant ING20160435478). We are grateful to François Lacoste for fruitful discussions and the Fonds Inkermann for financial support of the study. The authors also acknowledge the financial support from the Cnrs, Inserm, the Laboratoire d’Excellence GR-Ex (ANR-11-LABX-0051) and the Laboratoire d’Excellence ParaFrap (ANR-11-LABX-0024).

## Conflict of Interest

The authors declare that the research was conducted in the absence of any commercial or financial relationships that could be construed as a potential conflict of interest.

## Publisher’s Note

All claims expressed in this article are solely those of the authors and do not necessarily represent those of their affiliated organizations, or those of the publisher, the editors and the reviewers. Any product that may be evaluated in this article, or claim that may be made by its manufacturer, is not guaranteed or endorsed by the publisher.
