# Supplementary material for: The Phosphodiesterase Inhibitor Tadalafil Promotes Splenic Retention of Plasmodium falciparum Gametocytes in Humanized Mice
Source: Front Cell Infect Microbiol. 2022 May 25;12:883759. doi: 10.3389/fcimb.2022.883759 (PMC9174641; doi:10.3389/fcimb.2022.883759)
Supplement: Supplementary file 1 [file DataSheet_1.pdf]

The phosphodiesterase inhibitor tadalafil promotes splenic retention *of Plasmodium falciparum* gametocytes in humanized mice

Barbieri Daniela[1], Gomez Lina[1], Royer Ludivine[1], Dupuy Florian[1], Franetich Jean-François[2], Tefit Maurel[2], N'Dri Marie-Esther[1], Mazier Dominique[2], Silvie Olivier[2], Moreno-Sabater Alicia[2,3], Lavazec Catherine[1]

SUPPLEMENTAL FIGURES

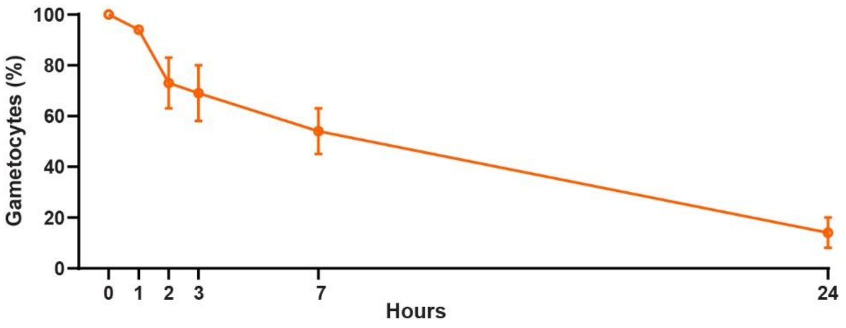

Supplemental Figure 1. Kinetics of mature GIE circulation in humanized mice during 24 hours.

Quantification of mature GIE clearance in peripheral blood during 24 hours post-injection by flow cytometry (n=2 mice). The percentage of gametocytes is normalized to the gametocytemia at 10 minutes after injection. Error bars show the SEM.

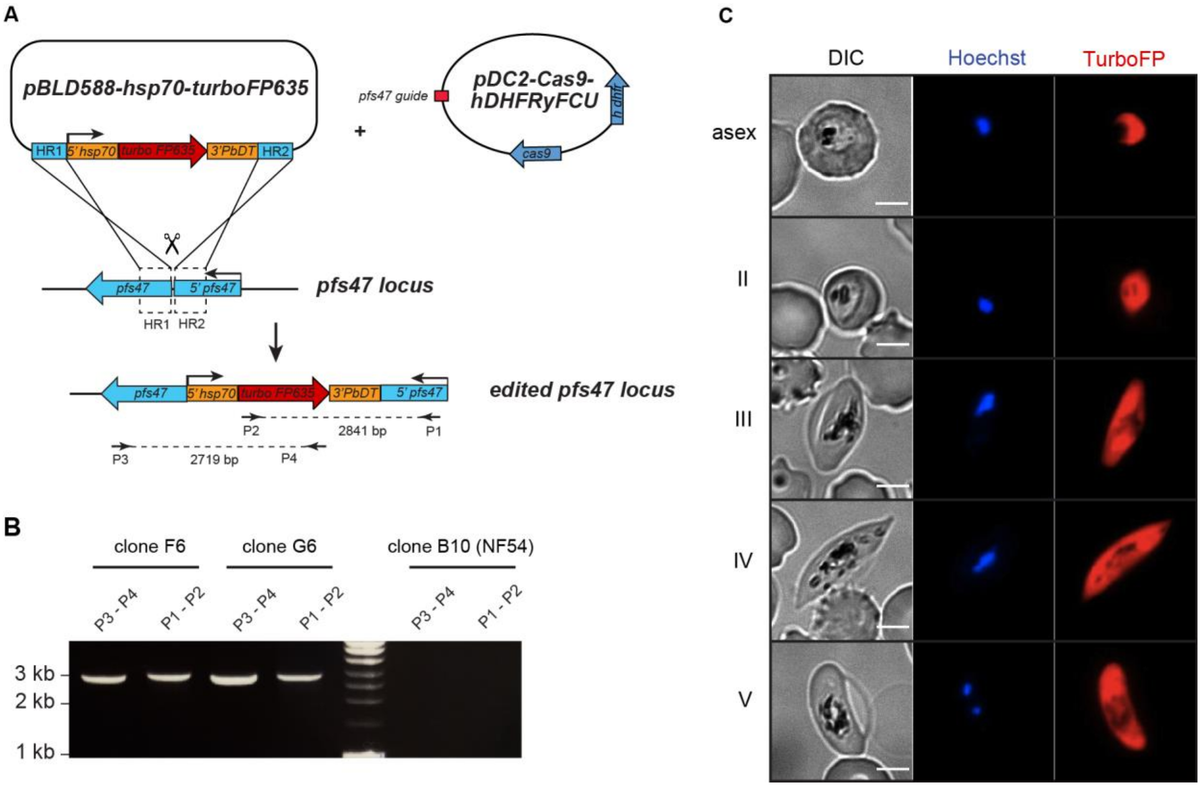

Supplemental Figure 2. Generation of parasites expressing TurboFP635 under the control of the hsp70 promoter using CRISPR-Cas9 technology

A. Schematic (not to scale) of the *turbofp635* insertion into the *pfs47* locus. The scissors indicate the Cas9 cleavage site. Arrows indicate the position of primers used to confirm plasmid integration into the *pfs47* locus by PCR. **B**. PCR analysis of genomic DNA from F6 and G6 clones obtained by limiting dilution of the NF54-pfs47-hsp70-TurboFP635 line. The NF54 clone B10 was used as control. **C**. Fluorescence microscopy analysis of the transfected parasites showing fluorescence (red) in asexual (asex) and gametocytes from stage II to V. DNA is stained with Hoechst 33342 (blue). Scale bars: 2 µm.

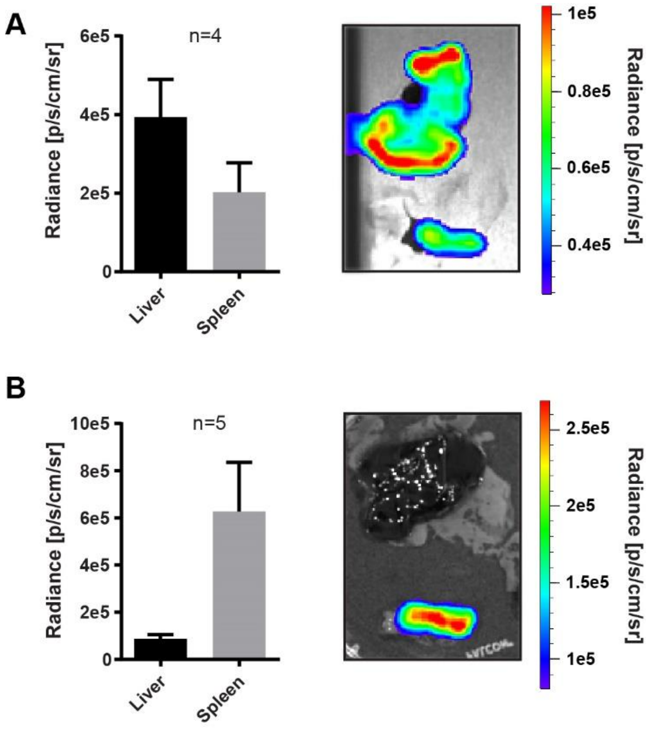

Supplemental Figure 3. Effect of hRBC engraftment on immature GIE sequestration in liver

A, B. Left: Quantification of GIE retention in liver and spleen visualized by measuring average radiance (p/s/cm^2/s) in dissected organs of ungrafted mice (A, n = 4 mice) and of hRBC grafted mice (B, n = 5 mice). Errors bars show the SEM. Right: Representative images of luminescent signals in dissected spleens and livers and heat map of radiance (p/s/cm^2/sr). Rainbow shows the relative level of luciferase activity.
